# What are the characteristics of people who use nicotine pouches and what types of pouches are being used? Data from an online cross-sectional survey of UK adults

**DOI:** 10.1371/journal.pone.0332962

**Published:** 2025-10-24

**Authors:** Katherine East, Harry Tattan-Birch, Eve Taylor

**Affiliations:** 1 Department of Primary Care and Public Health, Brighton and Sussex Medical School, University of Brighton and University of Sussex, Brighton, United Kingdom; 2 National Addiction Centre, Institute of Psychiatry, Psychology & Neuroscience, Kings College, London, United Kingdom; 3 Department of Behavioural Science and Health, University College London, London, United Kingdom; NYU Grossman School of Medicine: New York University School of Medicine, UNITED STATES OF AMERICA

## Abstract

**Introduction:**

Use of nicotine pouches is rising but little independent data exist on the types of pouches used by UK adults. This study therefore assesses: (1) demographic characteristics associated with using nicotine pouches; (2) nicotine content, flavours, brands, and frequency of use of nicotine pouches.

**Methods:**

Online cross-sectional Qualtrics survey of N = 2,967 UK adults (≥18 years) conducted May/June 2024. The sample were drawn from Prolific Academic using quotas (age, biological sex, ethnicity). Measures included demographics, vaping status, smoking status, frequency of nicotine pouch use, and pouch brand, flavour, and nicotine content used most often.

**Results:**

Overall, 2.9% (n = 85) currently used, and an additional 10.1% (n = 299) had tried, pouches. Ever use declined with age and was most common among males (AOR = 1.80, 95%CI = 1.40–3.31) and those who currently vaped (AOR = 7.19, 4.80–10.75) or smoked (AOR = 5.80, 3.82–8.80). Nicotine pouch use was rare among adults who never smoked (3.0%) or never vaped (2.7%). Among adults who had ever used nicotine pouches (n = 384), half used Nordic Spirit (49.7%), most used mint/menthol flavours (60.7%), the most popular nicotine content was 11–20 mg (34.6%) although many did not know the nicotine content (28.4%), and around a fifth (19.5%) reported using them every day, with an additional fifth (21.9%) a few times a week, and a quarter tried once or twice (25.5%).

**Conclusions:**

Among UK adults, nicotine pouches were predominantly used by those who currently/formerly smoke or vape and were also most popular among males and younger adults. Mint/menthol and 11–20 mg pouches were most popular and use frequency patterns varied substantially.

## Introduction

Oral nicotine pouches are small, permeable bags, typically filled with nicotine and other constituents such as flavourings, that are designed to be placed between the lip and the gum [1–3]. Unlike Swedish snus, they do not contain tobacco leaf. Nicotine pouches vary in nicotine content [2,3] and in the United Kingdom (UK) there is currently little specific regulation on nicotine pouches, meaning that there is no legal limit on the amount of nicotine in a pouch and no restriction on sales to children. However, the proposed 2024−25 Tobacco and Vapes Bill will include nicotine pouches under similar legislation to tobacco cigarettes and e-cigarettes, thus prohibiting their sale to under-18s and regulating advertising/sponsorship. The Bill will also provide the government with new powers to restrict the flavours, packaging, and display of nicotine products, as well as the amount of nicotine that may be included.

Pouches are currently advertised in the UK across many channels. including on social media, through motorsports sponsorship, at music concerns and festivals, and in train stations [4]. Nicotine pouches are also sold in a variety of flavours, such as fruit flavours, mint flavours, and spice flavours (such as cinnamon and chilli) [1,3]. Numerous brands also exist and almost all are owned by tobacco or vape companies (e.g., Zyn, Velo, On!, Nordic Spirit, Tacja) [3]. In 2023, the market share was dominated by Nordic Spirit and Velo [3,5], which are owned by Japan Tobacco International and British American Tobacco, respectively.

Use of nicotine pouches is rising. Online sales of nicotine pouches grew by 82% in 2023 [5] and the UK nicotine pouch market size was estimated at £217 million in 2023 and is predicted to rise further [6]. Among adults, in 2024, prevalence of current nicotine pouch use was 0.9% and prevalence of ever nicotine pouch use was 5.4%, both with increases since 2020 [7,8]. Nicotine pouch use is more common among men, younger age groups, people from London, people who currently or formerly smoke, people who vape e-cigarettes, and people who use nicotine replacement therapies [7,9].

Industry sales data from 2023 suggest that the most popular nicotine pouch brands were Velo and Nordic Spirit, followed by Loop, On!, and Zyn [5]. The most commonly purchased nicotine pouch flavours were mint followed by berry, fruit, and citrus, and ‘extra strong’ and ‘slim’ pouches were also commonly purchased [5]. To our knowledge there are currently no independent, published survey data in the UK on the types of nicotine pouches used, or the frequency of use, among adults in the UK. Research in this area is important in light of the proposed Tobacco and Vapes Bill, as it can help inform potential government restrictions on flavours, packaging, and nicotine content, which the Bill would grant new powers to enforce.

This study therefore addresses the following research questions:

1. What are the characteristics of adults (≥18 years) who have ever used nicotine pouches in the UK?
2. Among adults who have ever used nicotine pouches, what are the most popular types of products (nicotine content, flavours, brands) used and how frequently were pouches used?

## Materials and methods

### Design and sample

This work is a secondary analysis of data from an online survey assessing responses to nicotine pouch packaging [10]. The survey was hosted on Qualtrics and data were collected between 24 May and 6 June 2024. Participants were adults aged ≥18 years, drawn from Prolific Academic (a pre-existing panel) with quota samples set to ensure the sample was representative of age, biological sex, and ethnicity in the UK. All participants provided written informed consent. Ethical approval was granted by King’s College London (MRA-23/24–42484). Further details on the survey design are available on the Open Science Framework [10]. An initial sample of N = 3,025 was recruited, of whom 58 participants were removed due to missing demographic data, leaving an analytical sample of N = 2,967.

### Measures

The full list of measures is available on the Open Science Framework (https://osf.io/z3yfj) [10] and briefly described below.

#### Nicotine pouch use.

Participants were asked “Nicotine pouches are small pouches of nicotine which are placed in the mouth between your lip and gum. Brands include Lyft, Skruf, ZYN, Nordic Spirit and Velo. Which of the following statements BEST applies to you?”. Response options were: 1) I have tried nicotine pouches and still use them, 2) I have tried nicotine pouches but do not use them (anymore), 3) I have heard of nicotine pouches but have never tried them, 4) I have never heard of nicotine pouches, 5) Don’t know. Responses were coded as ‘Ever use (1-2)’ and ‘Never use/Don’t know (3-5)’. There were low sample sizes of participants who have tried nicotine pouches and still use them (i.e., current use; n = 85; S1 Table, Supplementary Materials) and so we did not explore this as a unique outcome in our analyses.

#### Nicotine pouch product and use characteristics.

Participants who reported that they had ever used a nicotine pouch were asked about their brand, flavour, and nicotine content used most often as well as their frequency of use.

#### Demographics.

Demographics were age group (18–24, 25–34, 35–44, 45–54, 55 + years), gender (male, female, in another way), perceived financial status (comfortable, coping, finding it difficult, finding it very difficult), and ethnicity (White, Asian, Black, Mixed, Other).

#### Vaping status.

Participants were asked about their use of vapes, with response options coded as: currently vape, vaped in the past, never vaped.

#### Smoking status.

Participants were asked about their use of cigarettes or other kinds of tobacco that are combusted, with response options coded as: currently smoke, smoked in the past, never smoked.

### Data analysis

Analyses were not pre-registered and so should be considered exploratory. Logistic regression analyses were used to explore the association between ever use of nicotine pouches as the outcome and participant demographics, vaping status, and smoking status as predictors. Predictors were entered separately into the models for unadjusted analyses and simultaneously for adjusted analyses. Frequencies and percentages were used to describe the characteristics of the nicotine pouches that people use. Data were not weighted. Analyses were completed using SPSS v28.

## Results

Overall, 28.4% (n = 842) of adults had never heard of nicotine pouches, 58.3% (n = 1730) had heard of pouches but had never used them, 10.1% (n = 299) had tried pouches but did not currently use them, 2.9% (n = 85) currently used pouches, and 0.4% did not know (n = 11) (S1 Table, Supplementary Materials).

Table 1 shows the proportion of adults who reported ever using nicotine pouches, by demographic and smoking/vaping subgroups. For both adjusted and unadjusted models, there were associations between having ever used a nicotine pouch and age group, gender, vaping status, and smoking status, such that ever use of nicotine pouches declined with age, was most common among those who identified as male, and those who currently vaped or currently smoked. Participants who currently vaped had seven times greater odds (AOR = 7.19, 95%CI = 4.80–10.75) of ever using pouches than people who had never vaped. Likewise, participants who currently smoked had six times greater odds (AOR = 5.80, 95%CI = 3.82–8.80) of ever using pouches than people who had never smoked. Differences in ever use of pouches by ethnicity or perceived financial status were not significant, but confidence intervals included the possibility of important associations.

**Table 1:** Sample characteristics and prevalence of nicotine pouch use by demographic and vaping and smoking subgroups among UK adults in 2024 (N = 2,967).

	Sample characteristics	Ever used a nicotine pouch				
	Row % (n)	Prevalence % (n)	Unadjusted		Adjusted	
			OR (95% CI)	p	AOR (95% CI)	p
**Ethnicity**						
White	86.0 (2553)	13.0 (331)	1.00 (ref)	**–**	1.00 (ref)	–
Asian, Asian British	7.8 (232)	14.2 (33)	1.13 (0.76-1.94)	.586	0.99 (0.63-1.56)	.958
Black, Black British, Caribbean, African	3.6 (106)	13.2 (14)	1.02 (0.58-1.81)	.942	0.83 (0.43-1.58)	.566
Mixed or Multiple ethnic groups	1.4 (43)	9.3 (4)	0.69 (0.24-1.94)	.480	0.64 (0.20-2.03)	.450
Other	1.1 (33)	6.1 (2)	0.43 (0.10-1.82)	.253	0.24 (0.05-1.10)	.066
**Age**						
18-24	10.6 (314)	21.3 (67)	1.00 (ref)	**–**	1.00 (ref)	–
25-34	17.2 (511)	15.3 (78)	**0.66 (0.46-0.95)**	**.027**	**0.49 (0.32-0.75)**	**.001**
35-44	16.3 (483)	16.1 (78)	0.71 (0.49-1.02)	.064	**0.57 (0.37-0.87)**	**.010**
45-54	17.2 (510)	12.5 (64)	**0.53 (0.36-0.77)**	**<.001**	**0.41 (0.26-0.64)**	**<.001**
55+	38.7 (1149)	8.4 (97)	**0.34 (0.24-0.48)**	**<.001**	**0.33 (0.21-0.50)**	**<.001**
**Gender**						
Female	51.5 (1527)	8.3 (127)	1.00 (ref)	**–**	1.00 (ref)	**–**
Male	48.0 (1425)	17.3 (254)	**2.39 (1.91-3.00)**	**<.001**	**1.80 (1.40-3.31)**	**<.001**
In another way	0.5 (15)	20.0 (3)	2.76 (0.77-9.89)	.120	2.06 (0.51-8.40)	.312
**Perceived financial status**						
Comfortable	26.6 (790)	11.1 (88)	1.00 (ref)	**–**	1.00 (ref)	–
Coping	47.8 (1419)	13.4 (190)	1.23 (0.94-1.61)	.127	0.99 (0.73-1.35)	.961
Finding it difficult	18.0 (534)	13.3 (71)	1.22 (0.88-1.71)	.237	0.75 (0.51-1.10)	.752
Finding it very difficult	7.6 (224)	15.6 (35)	1.48 (0.97-2.26)	.071	0.89 (0.55-1.44)	.893
**Vaping status**						
Never vaped	58.1 (1724)	2.7 (46)	1.00 (ref)	**–**	1.00 (ref)	**–**
Used to vape	24.8 (737)	21.0 (155)	**9.72 (6.90-13.68)**	**<.001**	**4.45 (3.05-6.51)**	**<.001**
Currently vape	17.1 (506)	36.2 (183)	**20.67 (14.64-29.17)**	**<.001**	**7.19 (4.80-10.75)**	**<.001**
**Smoking status**						
Never smoked	50.1 (1485)	3.0 (45)	1.00 (ref)	**–**	1.00 (ref)	**–**
Used to smoke	30.6 (909)	16.5 (150)	**6.32 (4.48-8.93)**	**<.001**	**3.94 (2.63-5.90)**	**<.001**
Currently smoke	19.3 (573)	33.0 (189)	**15.75 (11.17-22.22)**	**<.001**	**5.80 (3.82-8.80)**	**<.001**

Table 2 shows the product and use characteristics of people who had ever used a nicotine pouch. Around half (49.7%) of participants reported using Nordic Spirit, with Velo (18.8%) and Zyn (16.9%) being the second and third most popular brands. Over half used mint/menthol (60.7%), with tobacco (29.2%) and fruit (25.5%) being the second and third most popular flavours; few used another flavour or did not know what flavour they had used. A third (34.6%) of participants reported using a nicotine content of 11–20 mg and just under a third (28.4%) did not know the nicotine content of the pouch that they used. Around a fifth (19.5%) of those who had ever used a nicotine pouch reported using them every day, with an additional fifth (21.9%) reporting using them a few times a week and around a quarter reporting only having tried them once or twice (25.5%).

**Table 2:** Product and use characteristics of people who had ever used a nicotine pouch among adults in the UK in 2024 (N = 384).

	Row % (95% CI)	n
Brand*		
Nordic Spirit	49.7 (44.7-54.7)	191
Velo	18.8 (15.1-23.0)	72
Zyn	16.9 (13.5-21.0)	65
Lyft	14.3 (11.2-18.2)	55
Skruf	6.3 (4.2-9.2)	24
Elf/Tacja	3.6 (2.2-6.1)	4
Other	5.5 (3.6-8.3)	21
Don’t know	18.0 (14.4-22.2)	60
Flavour*		
Mint/menthol	60.7 (55.7-65.5)	233
Tobacco	29.2 (24.8-33.9)	112
Fruit	25.5 (21.4-30.1)	98
Other	2.1 (1.0-4.1)	8
Don’t know	7.0 (4.9-10.1)	27
**Nicotine content**		
1-10 mg	15.4 (12.1-19.3)	59
11-20 mg	34.6 (30.0-39.6)	133
21-30 mg	13.0 (10.0-16.8)	50
31-40 mg	4.4 (2.8-7.0)	17
41-50 mg	2.3 (1.2-4.5)	9
50 + mg	1.8 (0.9-3.8)	7
Don’t know	28.4 (24.1-33.1)	109
**Frequency of use**		
Every day	19.5 (15.9-23.8)	75
A few times a week	21.9 (18.0-26.3)	84
Once a week	7.8 (5.5-11.0)	30
Once or twice a month	8.3 (6.0-11.6)	32
Less than once a month	16.1 (12.8-20.2)	62
Have only tried once or twice	25.5 (21.4-30.1)	98
Don’t know	0.8 (0.3-2.4)	3

*Participants could choose multiple responses.

## Discussion

Consistent with national surveys of adults [7,9], our study found that nicotine pouch use was more common among men, younger age groups, and people who currently or formerly smoke or vape. Also consistent with national surveys, use was rare among people who have never smoked and or vaped [7,9]. Future research should investigate if pouches are being used for smoking and or vaping cessation, or as a substitute or supplement to these other forms of nicotine use.

Consistent with industry market data from 2023 [5], in 2024 the majority of adults who used nicotine pouches used mint flavour (61%) and the most popular brand was Nordic Spirit (50%). Market data show that “extra strong” pouches were the most commonly sold [5], which broadly aligns with our data suggest that the nicotine strength 11–20 mg was the most commonly used (35%)—pouches described as “extra strong” by Nordic Spirit and Velo are 11 mg and 18 mg, respectively. However, many people did not know the strength of the nicotine pouch that they used (28%); this may be because people who have used them only once or twice were unaware of the strength or because nicotine strength descriptors vary from brand to brand (e.g., “extra strong” can mean 9 mg for some brands and 60 mg for others). Standardising the description of nicotine pouch strength across brands might be helpful to enable consumers to make informed decisions about their use and reduce the potential for dependency. However, the nicotine content in the pouch does not necessarily directly relate to the amount of nicotine absorbed by the user. Pharmacological studies show that the nicotine delivery of pouches also depends on several other factors including the form of nicotine used and the wetness and alkalinity of the pouch [2,11]. Therefore, two different brands of pouches with the same nicotine content could deliver vastly different amounts of nicotine to the user [12].

Several different patterns of nicotine pouch use frequency emerged, with around a quarter of those reporting ever use having only tried nicotine pouches once or twice (26%) and around half reporting use at least once a week (49%). Additional research with larger samples is required to explore the characteristics of people who use nicotine pouches regularly and the motivations for these use patterns.

This study has limitations. First, although we used quota sampling to achieve a sample nationally representative of age, biological sex, and ethnicity, our sample was skewed towards people with higher socioeconomic status and prevalence of current smoking and vaping were higher than in national surveys [7,8]. Moreover, our estimates of ever (13.0%) and current (2.9%) use of nicotine pouches were higher than in national surveys from 2024 (5.4% and 0.9%, respectively [7,8]). These differences are likely explained by our sampling methods, whereby we recruited adults from Prolific Academic’s panel into a study assessing the impact of nicotine pouch packaging, which was likely to attract people who knew about, or use, these nicotine pouches. Therefore, findings should not be used as a population prevalence estimate of nicotine pouch use in the UK. Second, due to low sample sizes of people who currently used nicotine pouches, we could not explore the characteristics of this group. Additional studies with larger numbers of current users of nicotine pouches is therefore required. Third, the survey relied on forced-choice responses and did not collect qualitative data or data on motivations for use, which would have added additional depth and should be explored in future research.

This study also has strengths. To our knowledge, this is the first survey to provide data on the types of nicotine pouch products most commonly used by adults in the UK, as well as the characteristics of users. Our sample of 384 adults who had ever used nicotine pouches allowed us to explore brand, flavour, nicotine content, and frequency of use.

## Conclusions

To conclude, among a sample of adults from the UK, nicotine pouches were predominantly used by those who currently or formerly smoke or vape and were also most popular among males and younger age groups. Among adults who had ever used nicotine pouches, mint was used by the majority and the most popular nicotine content was 11–20 mg although many were uncertain about the nicotine content of their pouches. Frequency of use varied substantially, with around a quarter of adults who had ever used nicotine pouches having only tried them once or twice and around half reporting regular (i.e., at least weekly) use.

## Supporting information

S1 Table Sample characteristics by nicotine pouch use among UK adults in 2024 (N = 2,967). (DOCX)
