# Supplementary material for: What are the characteristics of people who use nicotine pouches and what types of pouches are being used? Data from an online cross-sectional survey of UK adults
Source: PLoS One. 2025 Oct 24;20(10):e0332962. doi: 10.1371/journal.pone.0332962 (PMC12551820; doi:10.1371/journal.pone.0332962)
Supplement: S1 Table — (DOCX) [file pone.0332962.s001.docx]

| **Table S1. Sample characteristics by nicotine pouch use among UK adults in 2024 (N=2,967).** | | | | | |
| --- | --- | --- | --- | --- | --- |
|  | **Never heard of nicotine pouches (n=842)** | **Heard of nicotine pouches but have never tried them (n=1730)** | **Tried nicotine pouches but do not use them (anymore) (n=299)** | **Tried nicotine pouches and still use them (n=85)** | **Don’t know (n=11)** |
|  | **Row % (n)** | | | | |
| **Ethnicity** |  |  |  |  |  |
| White | 28.7 (733) | 58.1 (1483) | 10.3 (264) | 2.6 (67) | 0.2 (6) |
| Asian, Asian British | 20.3 (47) | 64.7 (150) | 9.9 (23) | 4.3 (10) | 0.9 (2) |
| Black, Black British, Caribbean, African | 30.2 (32) | 53.8 (57) | 7.5 (8) | 5.7 (6) | 2.8 (3) |
| Mixed or Multiple ethnic groups | 32.6 (14) | 58.1 (25) | 4.7 (2) | 4.7 (2) | 0 (0) |
| Other | 48.5 (16) | 45.5 (15) | 6.1 (2) | 0 (0) | 0 (0) |
| **Age** |  |  |  |  |  |
| 18-24 | 11.5 (36) | 66.9 (210) | 13.1 (41) | 8.3 (26) | 0.3 (1) |
| 25-34 | 20.7 (106) | 63.6 (325) | 11.9 (61) | 3.3 (17) | 0.4 (2) |
| 35-44 | 29.2 (141) | 53.8 (260) | 12.2 (59) | 3.9 (19) | 0.8 (4) |
| 45-54 | 29.4 (150) | 58 (296) | 10.4 (53) | 2.2 (11) | 0 (0) |
| 55+ | 35.6 (409) | 55.6 (639) | 7.4 (85) | 1 (12) | 0.3 (4) |
| **Gender** |  |  |  |  |  |
| Female | 35.9 (548) | 55.4 (846) | 6.9 (106) | 1.4 (21) | 0.4 (6) |
| Male | 20.3 (289) | 61.5 (877) | 13.3 (190) | 4.5 (64) | 0.4 (5) |
| In another way | 33.3 (5) | 46.7 (7) | 20 (3) | 0 (0) | 0 (0) |
| **Perceived financial status** | |  |  |  |  |
| Comfortable | 32.5 (257) | 56.1 (443) | 7.5 (59) | 3.7 (29) | 0.3 (2) |
| Coping | 27 (383) | 59.3 (842) | 10.9 (154) | 2.5 (36) | 0.3 (4) |
| Finding it difficult | 26.6 (142) | 59.9 (320) | 11.2 (60) | 2.1 (11) | 0.2 (1) |
| Finding it very difficult | 26.8 (60) | 55.8 (125) | 11.6 (26) | 4 (9) | 1.8 (4) |
| **Vaping status** |  |  |  |  |  |
| Never vaped | 36.9 (637) | 59.9 (1032) | 2.6 (44) | 0.1 (2) | 0.5 (9) |
| Vaped in the past | 19 (140) | 59.7 (440) | 17.1 (126) | 3.9 (29) | 0.3 (2) |
| Currently vape | 12.8 (65) | 51 (258) | 25.5 (129) | 10.7 (54) | 0 (0) |
| **Smoking status** |  |  |  |  |  |
| Never smoked | 34.2 (508) | 62.2 (923) | 2.6 (38) | 0.5 (7) | 0.6 (9) |
| Smoked in the past | 26 (236) | 57.3 (521) | 14 (127) | 2.5 (23) | 0.2 (2) |
| Currently smoke | 17.1 (98) | 49.9 (286) | 23.4 (134) | 9.6 (55) | 0 (0) |
